# ‘Stumped’ by stump appendicitis—a case report and literature review

**DOI:** 10.1093/jscr/rjae573

**Published:** 2024-09-09

**Authors:** Chien Lin Soh, Shraddha Shetty, Sala Abdalla, Fiammetta Soggiu

**Affiliations:** Department of Surgery and Cancer, Imperial College London, Exhibition Rd, South Kensington, London SW7 2AZ, United Kingdom; Department of General Surgery, Ealing Hospital, London North West NHS Healthcare Trust, 601 Uxbridge Rd, Southall UB1 3HW, United Kingdom; Department of General Surgery, Ealing Hospital, London North West NHS Healthcare Trust, 601 Uxbridge Rd, Southall UB1 3HW, United Kingdom; Department of General Surgery, Ealing Hospital, London North West NHS Healthcare Trust, 601 Uxbridge Rd, Southall UB1 3HW, United Kingdom; Department of General Surgery, Ealing Hospital, London North West NHS Healthcare Trust, 601 Uxbridge Rd, Southall UB1 3HW, United Kingdom

**Keywords:** upper GI surgery

## Abstract

Stump appendicitis, a rare postoperative complication of appendicectomy, is inflammation of the remnant appendix tissue due to incomplete removal of the appendix at the index operation. Due to a past surgical history of appendicectomy, there is often a diagnostic delay. This delay can result in increased morbidity and mortality for patients. This series seeks to describe two cases encountered in a London district general hospital to elucidate the diagnostic, management, and operative challenges of stump appendicitis. Our case series demonstrates the importance of recognition of stump appendicitis as a differential for patients presenting with abdominal pain and previous appendicectomy. Active exclusion of this differential diagnosis in a patient with previous appendicectomy who presents with right iliac fossa pain is vital. Early identification and treatment can prevent morbidity in the patient population. We highlight that complete operative documentation and access to medical records are useful for this diagnosis.

## Introduction

Acute appendicitis remains one of the most common causes of attendance to the emergency department that culminates in urgent surgery [1, 2]. Stump appendicitis, a rare postoperative complication of appendicectomy, is inflammation of the remnant appendix tissue due to incomplete removal of the appendix at the index operation. The incidence is described in the literature as ranging from 0.002 to 0.15%, though it is estimated to be higher than previously reported [3]. Several factors are hypothesized to predispose to stump appendicitis such as the length of stump left in the index operation, difficult dissection or the presence of a faecolith [4].

Due to a past surgical history of appendicectomy, there is often a diagnostic delay. The clinical findings of stump appendicitis are similar to those of acute appendicitis - abdominal pain in the right lower quadrant, anorexia, and vomiting. This condition is often diagnosed with radiological imaging such as computed tomography (CT) scanning or ultrasonography [5, 6]. This presents a diagnostic challenge since stump appendicitis may not be recognized early, leading to delays in treatment and potential morbidity [7, 8].

This case series seeks to describe two cases encountered in a London district general hospital to elucidate the diagnostic, management, and operative challenges of stump appendicitis. It builds upon existing literature and highlights the importance of awareness of such condition. We highlight that complete operative documentation and access to medical records are useful for this diagnosis.

## Case 1: Patient A

Patient A, a 41-year-old male, presented to the emergency department with a history of generalized abdominal pain over 2 days with no inciting event. It was established from the history that the pain was acute in onset, sharp, and constant, with radiation to the right side of the back. The pain was exacerbated by movement and relieved by rest. He felt feverish with rigors and was constipated for 5 days with a complete loss of appetite. He did not experience any nausea, vomiting, or urinary symptoms. He had a past medical history of type 2 diabetes and hypercholesterolemia, and a history of appendicectomy 2 years prior to this presentation. Examination revealed a soft yet tender right upper quadrant with no peritonism. The patient was hemodynamically stable but had a low-grade fever of 37.6 °C. Urine dipstick was only positive for glucose. He had a normal white cell count however a C-reactive protein (CRP) of 37 (normal range < 2 g/dL). The initial chest X-ray was unremarkable with no free air under the diaphragm.

The working diagnosis was biliary colic and the patient was discharged with antibiotics after obtaining some pain control, with a planned review in the ambulatory care unit in 2 days. On review 2 days later, the patient’s symptoms had worsened and on examination there was now tenderness in the right flank and right renal angle, therefore the patient underwent a computerized tomography of the kidney, ureter and bladder (CT KUB) which revealed a thickened distal ileum, caecum and ileocecal junction, consistent with acute inflammation in the right iliac fossa.

Having had a previous appendicectomy with for perforated acute appendicitis with a postoperative collection, the differential diagnoses raised on CT KUB were either a stump appendicitis or a terminal ileitis. The patient was commenced on IV co-amoxiclav and formal contrast enhanced computer tomography of the chest, abdomen, and pelvis (CT CAP) was organized, revealing a residual long appendiceal stump infection associated with a small localized collection and caecal thickening.

After informed consent, the patient underwent a diagnostic laparoscopy with the intraoperative finding of multiple dense adhesions with the anterior abdominal wall that precluded safe approach to the right iliac fossa laparoscopically. After conversion to lower midline laparotomy and adhesiolysis, the inflamed stump was identified and the appendicectomy was completed with ligation of the appendiceal base at the convergence of the taenia coli. The postoperative course was uneventful, and the patient was discharged on postoperative Day 4.

The final histopathological examination revealed appendicitis, with an 80 mm length and 10 mm diameter with an attached mesoappendix. Microscopic analysis revealed mucosal ulceration and dense transmural neutrophilic inflammation and luminal fibrous obliteration.

**Figure 1:**
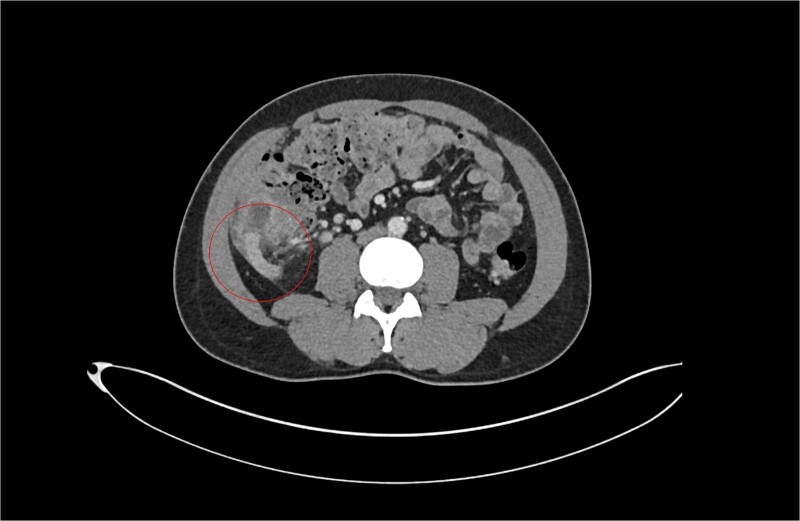
Patient A. Appendiceal stump of Patient A identified within the red circle on CT scan before the second operation.

Review of previous notes revealed that the patient had a laparoscopic appendicectomy 2 years prior from a CT-confirmed acute retrocaecal appendicitis with localized perforation at the tip (Fig. 1). Intraoperatively, the dissection proved difficult due to the presence on multiple inflammatory adhesions and the retrocaecal position of the appendix, however a retrograde appendicectomy was completed laparoscopically. The postoperative period was complicated by a right iliac fossa abscess that was successfully treated with IV antibiotics and CT-guided drainage. The index histology revealed multiple pieces of appendiceal tissue aggregating to 60 mm × 40 mm × 20 mm, with acute inflammation and necrosis.

## Case 2: Patient B

Patient B, an 18-year-old male, presented to the emergency department with sudden onset cramping generalized abdominal pain radiating to the right iliac fossa and testicle. This pain was notably described by the patient as similar to ‘appendicitis pain’ he experienced a few months ago, which culminated in an emergency appendicectomy 2 months prior to this presentation. Examination revealed guarding and tenderness in the right iliac fossa. McBurney’s sign was positive. There was no peritonism. Testicular examination was normal with no tenderness or swelling, and no clinical concern for torsion. He had a low-grade fever but was hemodynamically stable. Urine dipstick revealed blood in the urine. Blood tests revealed neutrophilia of 13.1 and CRP of 5.9*.*

**Figure 2:**
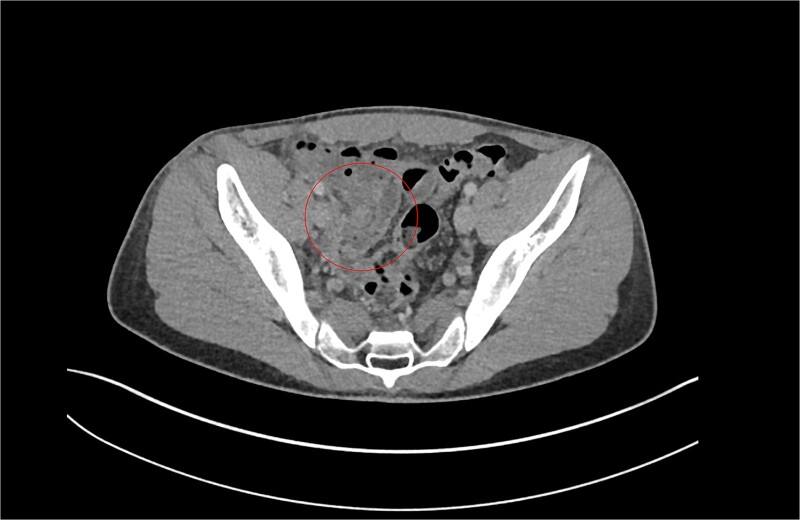
Patient B. Appendiceal stump of Patient B identified within the red circle on CT scan before the second operation.

With a differential diagnosis of stump appendicitis versus nephrolithiasis, a CT KUB was done which revealed no significant findings in the appendix or kidneys—the differential was revised to mesenteric adenitis or inflammatory bowel disease. The patient was counseled to be booked for an outpatient colonoscopy. However, in view of ongoing symptoms, serial examinations and investigations revealed a rising white cell count and CRP to 264. The patient was started on intravenous (IV) antibiotics. A formal CT CAP demonstrated mural thickening and enhancement of the caecal pole and fat stranding (Fig. 2). With a diagnosis of stump appendicitis, IV antibiotics were escalated to metronidazole and piperacillin-tazobactam.

The patient was counseled and consented for a diagnostic laparoscopy and completion appendicectomy. The caecum was mobilized from adhesions to the lateral abdominal wall and the convergence of taenia coli was followed to the appendicular stump. The operation revealed an acutely inflamed long retrocaecal appendiceal stump of ~3 cm length, with pus. Loose suture material was found in the vicinity of the stump. The remainder of the caecum and terminal ileum appeared normal. The patient had an uncomplicated recovery and was discharged 3 days later. Histological examination showed an appendix stump that was 3 cm long with chronic inflammation. There was no obvious malignant activity or periappendicitis.

Patient B had an uncomplicated laparoscopic appendicectomy 2 months prior in a different hospital. He presented to the hospital with fever, right iliac fossa pain and positive Rovsing’s sign. His blood tests revealed a raised white blood cell count of 14.7 and a neutrophilia of 11.65. The measured CRP was 5. The CT report described acute uncomplicated appendicitis with an associated large faecolith at the appendix base. He was treated with IV antibiotics and a same day laparoscopic appendicectomy, which was described as uncomplicated. The histology report revealed acute suppurative appendicitis with no parasites. The appendix measured 48 × 15 × 8 mm with an attached mesoappendix measuring 25 × 15 mm.

## Discussion

This case series seeks to describe the rare but challenging diagnosis of stump appendicitis in patient who present to the emergency department with right-sided abdominal pain on a background of previous appendicectomy.

This case series aims to highlight that appropriate clinical examination, urgent radiological imaging, and prompt surgical intervention are vital for good clinical care. However, there remains a diagnostic dilemma with no consensus on the ideal investigations or surgical approach nor on the risk factors on developing this delayed complication through national or international guidelines. Our case series aims to increase awareness of the condition with the hope of reducing diagnostic delay and encouraging timely intervention.

There have been previous case reports and literature reviews written about the subject of stump appendicitis [4, 5, 9–15]. The incidence of stump appendicitis is thought to be higher than previously documented [4]. Stump appendicitis may lead to the same complications of acute appendicitis including perforation, peritonitis, and septic shock, with significant risks of poor outcomes if the diagnosis is overlooked or delayed.

History taking in all patients revealed generalized abdominal pain that may or may not have radiated to the right iliac fossa—these signs unfortunately are nonspecific. All of our patients presented with neutrophilia and a rise in the CRP. With advancements in radiological imaging and the changing landscape of clinical practice, more patients with undifferentiated abdominal pain are referred for early imaging. Due to the diagnostic uncertainty and the concomitant rise in inflammatory markers, all patients in this case series had a CT scan for confirmation, however, imaging modalities such as abdominal ultrasound or MRI can also be utilized. CT scan with contrast was successful for identifying the inflamed appendiceal stump, however the findings may mimic those of acute appendicitis such as thickening of caecal wall, fat stranding, or localized fluid collection [16] (Table 1).

**Table 1:** Case summary describing patient demographics, primary diagnosis, complications, and time to surgery and discharge.

Case	Demographics	Case summary	Time to surgery from presentation to hospital	Time between initial Op and reoperation	Time to discharge	Complications
A	41M, type 2 diabetes mellitus, hypercholesterolemia	CT proven acute appendicitis	2 days	2 years	6 days	Postoperative pain
B	18M	CT proven acute appendicitis	3 days	2 months	6 days	

Management of stump appendicitis can be complicated by the prior surgery. Patient A had to be converted to open surgery due to intra-operative challenges such as significant adhesions and difficulty identifying the base of the appendix. However, Patient B had laparoscopic surgery. Both patients had initial laparoscopic appendectomies that may have been complicated, however, were successful through consistent identification of the stump and judicious dissection of adhesions. The operating surgeons were different for each operation within this case series which may have had an impact on surgical technique during the operation.

Risk factors for developing stump appendicitis include technical aspects of the index appendicectomy such as the lack of correct identification of the base of the appendix or its retrocaecal position that leads to a more difficult exposure. The presence of peritonitis, perforation, and adhesions can also increase the chances of not identifying the base. This is a critical step of laparoscopic appendicectomy, and Subramanian and Liang discuss a ‘critical view’ much like that of laparoscopic cholecystectomy that can prevent conversion to open surgery. The identification of the appendix, taenia caecum, and the terminal ileum is required to confirm position of the stump [13]. Another risk factors could be the length of stump left in the first surgery. Burbano et al. describe a stump as long as 7 cm being left behind [4]. In Patient A, the length of stump remaining was 8 cm, which is significant. General recommendation is that the appendix should be resected completely, with a stump <3 mm in length. Primary laparoscopic approach was initially thought to be a contributing factor due to lack of tactile response, however this school of thought remains controversial and not supported by the literature [17].

Stump appendicitis is usually treated with a surgical intervention. While there is evidence that non complicated acute appendicitis may be treated conservatively with antibiotics, no such strong evidence is available for stump appendicitis. A literature review from 2011 showed that out of 40 cases of stump appendicitis, all were operated on, and only 33% were managed laparoscopically [18]. In patients where operative management may not be the appropriate option, conservative management with IV antibiotics as described by Paudyal et al. has shown clinical effectiveness [19].

Time between primary surgery and re-do appendicectomy in our study ranged from 2 months to 2 years—this has been described in the literature as ranging from 4 days to 50 years [7, 17, 18].

Another challenge we identified in our case series was the lack of comprehensive original operative notes when the index surgery is performed at a different hospital. Clear intra-operative documentation and discharge documentation is essential to this diagnosis. There was difficulty in obtaining documentation regarding the first operation for Patient B due to different hospitals for each operation. Therefore, judicious communication between surgeons and hospitals is required to support the diagnosis and decision-making regarding surgery. This was a limiting factor in our ability to ascertain the exact surgical techniques and findings of the index operations—highlighting the need for further research into operative techniques to prevent stump appendicitis.

In conclusion, our case series demonstrates the importance of recognition of stump appendicitis as a differential for patients presenting with abdominal pain and previous appendicectomy. Active exclusion of this differential diagnosis in a patient with previous appendicectomy who presents with right iliac fossa pain is vital. Early identification and treatment can prevent morbidity in the patient population. Surgeons must take note of the importance of complete operative documentation, particularly the difficulties encountered in the previous surgery as these can provide valuable clues to the cause for the current presentation.
